# What if we decided to take care of everyone who needed treatment? Workforce planning in Mozambique using simulation of demand for HIV/AIDS care

**DOI:** 10.1186/1478-4491-6-3

**Published:** 2008-02-07

**Authors:** Amy Hagopian, Mark A Micek, Ferruccio Vio, Kenneth Gimbel-Sherr, Pablo Montoya

**Affiliations:** 1 Departments of Health Services and Global Health, University of Washington School of Public Health and Community Medicine, 4534 11th Av. NE, Seattle, WA 98105, USA; 2 Health Alliance International, Seattle, Washington, USA; 3 Health Alliance International, Maputo, Mozambique; 4 Health Alliance International, Beira, Sofala, Mozambique

## Abstract

**Background:**

The growing AIDS epidemic in southern Africa is placing an increased strain on health systems, which are experiencing steadily rising patient loads. Health care systems are tackling the barriers to serving large populations in scaled-up operations. One of the most significant challenges in this effort is securing the health care workforce to deliver care in settings where the manpower is already in short supply.

**Methods:**

We have produced a demand-driven staffing model using simple spreadsheet technology, based on treatment protocols for HIV-positive patients that adhere to Mozambican guidelines. The model can be adjusted for the volumes of patients at differing stages of their disease, varying provider productivity, proportion who are pregnant, attrition rates, and other variables.

**Results:**

Our model projects the need for health workers using three different kinds of goals:

1) the number of patients to be placed on anti-retroviral therapy (ART),

2) the number of HIV-positive patients to be enrolled for treatment, and

3) the number of patients to be enrolled in a treatment facility per month.

**Conclusion:**

We propose three scenarios, depending on numbers of patients enrolled. In the first scenario, we start with 8000 patients on ART and increase that number to 58 000 at the end of three years (those were the goals for the country of Mozambique). This would require thirteen clinicians and just over ten nurses by the end of the first year, and 67 clinicians and 47 nurses at the end of the third year. In a second scenario, we start with 34 000 patients enrolled for care (not all of them on ART), and increase to 94 000 by the end of the third year, requiring a growth in clinician staff from 18 to 28. In a third scenario, we start a new clinic and enrol 200 new patients per month for three years, requiring 1.2 clinicians in year 1 and 2.2 by the end of year 3. Other clinician types in the model include nurses, social workers, pharmacists, phlebotomists, and peer counsellors. This planning tool could lead to more realistic and appropriate estimates of workforce levels required to provide high-quality HIV care in a low-resource settings.

## Background

AIDS treatment advocates have finally prevailed in the public health debate about whether it is cost-effective or appropriate to launch wide-scale treatment programs for high-prevalence populations in low-income countries. The initial years of the global AIDS epidemic were characterized by both poor treatment options and an emphasis on prevention. Political activists and public health professionals are now on the same page: it makes sense to aggressively treat populations for AIDS using established World Health Organization (WHO) protocols, as both a preventive strategy and a way to mitigate the devastating effects of the disease on a primarily young population [1]. We have collectively turned our attention to the range of practical issues related to efforts to ensure universal access to treatment.

Pilot programs have long-since demonstrated that we can deliver appropriate treatment protocols and procedures. Now, public health and health care systems are tackling the barriers to serving large populations in scaled-up operations. One of the most significant challenges in this effort is securing the health care workforce to deliver the care in settings where the manpower is already in short supply [2,3].

This paper describes a simple spreadsheet-based model designed for use by Ministries of Health or other parties planning large-scale AIDS treatment programs to estimate personnel needs. Our model allows the user to change a series of assumptions to estimate the impacts of various 'what if' scenarios. This model is based on the situation in Mozambique, in sub-Saharan Africa, but was designed to be generalizable to any low-resource setting attempting to estimate the workforce needs of scaling up for AIDS treatment.

There is little in the literature on health workforce modelling techniques for resource-poor settings, especially on means for estimating requirements based on patient demand. Most health workforce planning uses practitioner-to-population ratios, historical patterns, and professional judgment. More sophisticated analyses may allow estimating workforce size and mix through use of case-load profiling, acuity measures, or a combination of factors in regression analysis [4,5]. Dreesch, Dolea et al. have developed an approach to estimating human resource requirements based on time needed to address health deficits of the population [5]. An unpublished 2004 WHO model is an exception to this, as it offers a 'user guide' to increasing access to anti-retroviral therapy (ART) by assessing health workforce needs [6].

Hirschhorn and colleagues, in estimating workforce needs for AIDS treatment in resource-limited settings, suggest a task-based approach to "explore the effect of reassigning tasks to other cadres" of health personnel [7].

Larry Faulkner has written about estimating psychiatric workforce requirements based on patient needs, and offers a simple formula for making calculations: (# patients needing care × amount of treatment time required)/amount of time offered per psychiatrist = number of psychiatrists required) [8].

### The Challenge in Mozambique

Mozambique's 2006 HIV prevalence among 15–49 year olds is extrapolated from 2004 figures, and stands at 16%, with 1.65 million adults and children living with HIV/AIDS [9]. The 2006 WHO AIDS epidemic update noted that Mozambique shows a significant increase in HIV infection levels since the turn of the century, and that prevalence in pregnant women (15–49) rose from 11% in 2000 to 16% in 2004, one of the steepest increases seen in sub-Saharan Africa in recent years [9]. Rising prevalence in pregnant women may suggest that new infections continue to increase, a signal of further growth in the epidemic in Mozambique [9].

It is estimated that over 270 000 Mozambicans were clinically eligible (under WHO guidelines) to receive ART in 2006. The country's National AIDS Strategic Plan (2004–2008) aimed to enrol more than 34 300 people for care in 2004, and more than 67 000 by 2005 [10] (See Table 1). In addition to those enrolled for care who were not yet eligible for ART, the plan called for 8000 people to be on ART by the end of 2004 and 21 000 people by the end of 2005. The Ministry of Health is reporting that as of December 2006, over 160 000 individuals had been enrolled for care (66 000 more than anticipated), and 44 100 were receiving ART (14 000 fewer than had been hoped).

**Table 1:** Mozambique strategic plan ART enrolment targets by fiscal year (2004–2008)

**Enrolment level**	**2004**	**2005**	**2006**	**2007**	**2008**
ART drugs	7 924	20 805	57 954	96 418	132 280
Care w/o drugs yet	34 311	67 779	94 178	108 207	114 965

The United States Agency for International Development (USAID) reported in 2004 that the health sector in Mozambique faced enormous challenges, including weak health infrastructure, significant budgetary constraints, endemic poverty in the population, old and emerging diseases, and poor health indicators [11]. The report expressed particular alarm about the threat of HIV/AIDS, both for the population in general and to the workplace in particular.

### Delivering HIV/AIDS Care in Mozambique: what staffing model works best?

After starting with a fairly vertical 'day hospital' model for HIV/AIDS patients, Mozambique has now moved to integrate its HIV care into the existing health system. The HIV integrated health network links voluntary counselling and testing (VCT), prevention of mother to child transmission (PMTCT), home-based care, and outpatient and inpatient care.

We searched for existing guidance on staffing models for Mozambique's ART initiative. USAID in 2004 had suggested staffing norms that seemed to be fairly generic and minimal [12]. These called for 1 physician and 3 nurses per health facility without any regard for patient volumes or treatment protocols. The report did note that varying staffing models are in place from site to site, and called for clarifying the treatment model before "projections of overall human resources requirements for the ART scale-up are worked out."

In a document dated about the same time, the Mozambique Ministry of Health called for a different mix of staff (1 physician, 1 nurse and 1 mid-level provider or medical technician), but again with no reference to the number or types of patients to be served with this staffing model.

Neither staffing standard takes into account the varying numbers or stages of disease of the patients using the over 150 health facilities providing ART, nor do they differentiate between fixed and variable staffing requirements. A minimum fixed number of administrative staff is required to organize care and systems, yet the number of these individuals required should be fairly independent of volumes (or would only change in large incremental blocks of patient volumes). A number of essential personnel categories, such as laboratory technicians, are not included at all.

### Available workforce for HIV care is small, ratios are high

The number of people with HIV/AIDS divided by the number of physicians in Mozambique indicates each physician needs to care for an average of 2155 HIV-positive patients. The averages change, however, by urban or rural status: physicians in Maputo City could be assigned 342 patients, while Zambesia-based physicians each have 6496 patients. This compares, for example, to a full-time HIV care provider in the US, who can be asked to carry a patient load of about 350 patients (personal communication, Robert Harrington, MD, medical director of the University of Washington/Harborview Medical Center HIV Clinic). The numbers in Mozambique indicate that rural physicians have a patient load that exceeds any reasonable standards, and even the expectations of the 'average' physicians are extremely high, especially if one adds in patient care and administrative responsibilities outside of caring for patients with HIV/AIDS. Mid-level health workers have the authority to prescribe ART in Mozambique.

This paper proceeds in the order we used to develop the model: 1) we discuss the assumptions, 2) we present the model (created in an Excel© spreadsheet, see Additional File 1), 3) we discuss the results, and, finally, 4) we discuss implications and conclusions.

## Methods

We produced a demand-driven staffing model using simple spreadsheet technology, based on treatment protocols for HIV-positive patients that adhere to Mozambican guidelines. As such, it represents the minimum requirements to successfully complete protocols for HIV treatment and accounts for some, but not all, the extra encounters that could be generated by complications such as opportunistic infections. The user can easily adjust for the volumes of patients at differing stages of their disease, varying provider productivity, proportion who are pregnant, attrition rates, and other variables.

### Assumptions

We relied on the document, "Human Capacity Development Assessment and Strategy Development for the Health Sector in Mozambique," previously referenced, and prepared by the Africa Bureau of USAID for some of our assumptions. This document will be referred to as the 'USAID document.' We also relied on the personal knowledge of four authors: Mark Micek, a physician with Health Alliance International (HAI), who was involved in the implementation of public-sector HIV treatment clinics in Beira and Chimoio, Mozambique; Kenneth Gimbel-Sherr, who is HAI's Mozambique country director and was involved in developing the original national plan with the Ministry of Health for providing HIV treatment nationwide; Ferruccio Vio, who works as Maputo Technical Support Coordinator for HAI; and Pablo Montoya, Central Mozambique Field Director for HAI, where he supports provincial planning for the Ministry of Health.

### Demand

We assumed patients present to the health care system following a referral from one of a number of HIV testing sites within a community, including VCT (voluntary counselling and testing) centres, PMTCT (prevention of maternal to child transmission) centres, and hospitals, so they are known to be HIV-positive upon arrival. Patient CD4 (Cluster of Differentiation 4) counts, however, are unknown at the time of presentation.

Our model allows the user to input the distribution of patients eligible for ART at their initial contact with the clinic. We assumed that 45% of the HIV-positive adult patients will need to be placed on anti-retrovirals upon presentation, and that another 5% of HIV-positive presenters will be pregnant women who would also benefit from immediate ART treatment. For those not initially eligible for ART, we estimated that an additional 20% would have initial CD4 counts from 200–349/mm^3^, 15% would have CD4 counts between 350–499/mm^3^, and 15% would have CD4 counts ≥500/mm^3^. These estimations are based on the experience at Beira and Chimoio, where it is notable that the proportion of people needing ART exceeds UNAIDS and strategic plan assumptions. Patients presenting for HIV care may not represent a cross-section of all HIV-positive patients, but rather those who sought testing and successfully presented for care at an HIV treatment facility referral. The population who seek testing may be in the more advanced stages of illness than those who postpone. It should be noted, however, that the model allows these assumptions to be changed depending on differing experiences at different sites. At this stage, we are not including children in the analysis.

Our demand-driven model also provides the user with an opportunity to input a variety of additional assumptions, including 1) the proportion of those eligible for ART who would start treatment (we assumed 70%), 2) the proportion who will leave the care system secondary to death or loss to follow-up (we assumed 10% per year for those on ART, 50% for those enrolled but not eligible for ART), 3) those with adverse drug reactions (we assumed 10%), and 4) those who experience a lack of clinical improvement and therefore may require more encounters (we assumed 10%).

We assumed patients start ART according to Mozambique Ministry of Health guidelines, which include all patients with CD4 levels under 200 cells/mm^3 ^regardless of clinical stage, a CD4 level between 200 and 349 cells/mm^3 ^if also in WHO stage 3 or pregnant, or WHO stage 4 regardless of CD4 count.

### Schedule of encounters

Our approach was to identify several 'types' of patients, and to map out the appropriate schedule of encounters for each newly-presenting type of patient based on published Mozambican guidelines [12].

'Encounters' in our model are from the care provider's point of view. A single patient trip to the clinic could generate several encounters if the patient sees more than one provider type during that trip. We will distinguish, therefore, between trips and encounters.

The first two patient trips consist primarily of assessment and planning procedures (including obtaining CD4 counts), so these would be the same for everyone. Trip 1 generates one encounter with a nurse for a clinical evaluation, and a separate encounter with a nurse who does a blood draw. This sample is sent to the lab, which takes 7 days to process and receive results.

Trip 2 is a week later than the first, at which time there is a single encounter with a nurse who evaluates the result of the CD4 count and makes a staging decision about the progress of the disease. This places the patient in one of several categories based on initial CD4 counts and clinical staging and on estimations about the rates in which people may change clinical categories over the duration of the 3 years of the model.

The patients who would need ART immediately would have an accelerated schedule of encounters: trip 3 would be within a week of the second trip and would generate two encounters: one with a clinician authorized to prescribe ART (mid-level medical technicians have been authorized to prescribe ART since June of 2006), and an encounter with a social worker to review the care plan. For these patients, trip 4 is with a social worker, as Mozambique recommends three encounters with a counsellor before ART is initiated. At trip 5, the patient is started on ART. There are encounters with the social worker, pharmacist and a clinician to discuss how the drugs will be administered and how to take them. At trip 6, two weeks after starting ART, there are encounters with a phlebotomist for haemoglobin and liver tests, a clinician, a pharmacist, and a counsellor to assess the course of therapy and review blood work results. Subsequently, these patients have monthly encounters to a pharmacist, and will see a clinician and counsellor at months one, two, four, seven and ten after starting ART. For pregnant women, encounters with a phlebotomist (for haemoglobin) and a clinician are also required at six weeks to monitor the side effects of AZT. Routine CD4 counts at month four and every six months thereafter require a trip and a encounter for blood draws. For each cohort starting ART, we estimate that 10% will have significant reactions or illnesses during the initial two months of treatment that will require further clinical encounters. In addition, at each CD4 draw time, we estimate 10% of patients will be identified as potential treatment failures, and will require additional encounters that are included in our model. Again, these assumptions are modifiable depending on differing experiences encountered at different sites.

For those patients who are not yet eligible for ART, we scheduled nursing encounters to repeat CD4 testing at intervals specified by Mozambique recommendations. This includes encounters every three months for those whose CD4 counts are between 200 and 349; encounters every 6 months for those with CD4 counts between 350 and 499, and encounters every 12 months for those with CD counts at or above 500.

To estimate encounters in the second and third years of follow-up for patients not initially ART-eligible, we estimated the proportion of patients presenting in each of the of clinical stages, and the rates at which these people may change clinical categories over the duration of the 3 years of the model. The encounter schedule will change, therefore, based on the progressing clinical stage.

### Supply

The USAID document (p. 21) states that physicians can be expected to work 1600 hours per year, or 200 working days at eight hours per day. This assumes about 40 weeks of work a year, or significantly below typical U.S. working expectation of 45 to 48 weeks. Furthermore, the document discounts those 1600 hours by an additional 20% (ostensibly, but not explicitly, for administrative time) to yield 1280 patient contact hours per year. With 1280 contact hours per year over 200 days, or 6.4 hours per day, we calculate an encounter takes 12 minutes and that a clinician can be assigned roughly 6000 encounters per year.

Our model assumes nurses can be assigned 6000 encounters per year. A social worker would be able to complete 3000 encounters, and a pharmacist could process 10 000. Peer counsellors, or activists, are projected to be able to follow up five missing patients per day, and need is projected by multiplying 15% times the number of pharmacy encounters. A phlebotomist would be able to draw blood on 25 patients per day, or 5000 patients per year. Changing our productivity assumptions would change our staffing requirements, of course.

The same document estimates there were 647 physicians in Mozambique at the end of 2003, about 40% of whom were specialists. A draft Human Resources Development Plan calls for that number to more than double by 2010. This plan further calls for an additional 1255 nurses beyond the 4025 estimated to be practicing in 2004 [13].

In addition to the small numbers of personnel, other supply problems named include weak human resource management, too few administrative managers, low motivation levels of health workers, high turnover or loss of health workers secondary to HIV-related or other serious illness, and a shortage of protective equipment and supplies. Our report does not address these issues.

### Model

There are nine categories of health worker in our model. These include:

1) Adult non-obstetrical (non-OB) clinicians (physicians and ARV-trained mid-level medical technicians), trained to make decisions about ART therapy;

2) Adult non-OB clinicians, who can manage ART therapy, but are not trained or required to make decisions regarding starting or changing ART regimens, and do not need to be a physician or ART-trained mid-level medical technician;

3) Obstetrical clinicians, trained to both start ARTs and manage them through the patient's pregnancy;

4) Obstetrical clinicians who can manage ART therapy but are not trained or required to make decisions regarding starting or changing ART regimens;

5) Clinical nurses who can evaluate CD4 counts and make referrals to clinicians for ART therapy;

6) Phlebotomists, who can draw blood samples for CD4 and other blood tests and send them to laboratories for processing;

7) Social workers, who engage in pre- and post-ART counselling;

8) Pharmacists, who dispense ART drugs; and

9) Lay peer-counsellors (Activistas), who are an important component of the health care team but whose roles are not well defined. These individuals are responsible for finding patients who seem to be lost to follow up, and can also support chart management and receptionists and perform other all-around tasks, such as adherence counselling, patient orientation, and HIV prevention counselling.

Our model consists of four interconnected spreadsheets that build on each other but are relatively simple to understand. See Additional File 1 for a live spreadsheet workbook.

The fundamental spreadsheet (Worksheet A) uses the individual patient as the unit of analysis. It has 36 columns – one for each month of 3 years. The rows are grouped into five categories of patient type based on their health status at the time of enrolment: 1) those who need ART now, 2) those with a low CD4 count (200–349) who will need ART 'later' (within 1 year after enrolment); 3) those with a low CD4 count (200–349) who will not need ART within 2 years, 4) those who have CD4 counts of between 350 to 499, and 5) those whose counts are at or above 500. These numbers are automatically generated based on the assumptions entered in the 'Assumptions' section of Worksheet D.

Worksheet A maps out the encounters described in schedule of encounters,' above, over a three-year period.

The next spreadsheet (Worksheet B) also has 36 columns (one for each month), but the rows consist of total patient encounter counts generated in the patient-level Worksheet A, depending on the number of patients entered into the system. These total counts are also grouped by category of patient. The patient attrition assumptions (entered by the user) are played out in this spreadsheet.

The third spreadsheet (Worksheet C) is a large one, and is from the care system's point of view. Monthly encounter counts generated by type of patient are totalled and scheduled over a three-year period.

The initial "input control" spreadsheet (on the same page as Worksheet D) allows users to enter assumptions about patient distribution characteristics as well as to select one of three patient volume scenarios. Method 1 allows the entry of a total number of people on ART in the system at three time points one, two and three years after 'time zero'. Method 2 allows the entry of a total number of people for care (whether or not they are on ART) in the system at the one, two and three year time points after 'time 0' (these can be at either the clinic or national level). Method 3 allows the entry of a number of patients enrolled in a care system per month (at either the clinic or national level), and is intended to represent the care system's flows during a steady state period. Only one method at a time may be used. At this time, the model looks forward for only a three-year period.

When patient volume inputs are entered, a 'Summary Table' on the worksheet calculates numbers of patients enrolled per month for each of the three years in the model.

## Results

### Three scenarios generate different staffing configurations

Using each of the methods offered by the Input Control portion of the model, we offer three scenarios and sets of results by way of example. After inputting assumptions and patient enrolment goals, we see results displayed in the 'summary' tab of the Excel^© ^workbook.

### Scenario #1

We start year one with no one yet on ART and grow to 8000 starting ART by the end of year one. (for Mozambique, this was 2004). We reach the Mozambique goal of 21 000 individuals starting ART at the end of year two, and grow to 58 000 by the end of year three. See Table 2.

**Table 2:** Results of our model, Scenario 1: 8000 starting ART by the end of year 1, ending with 58 000 starting ART by the end of year 3

Clinician type*	**End year 1**	**End of year 3**
Number of non-OB encounters with clinicians	5753/month	30 089/month
OB encounters with clinicians**	712/month	3428/month
FTEs required to meet this demand based on our productivity assumptions	11.5 non-OB clinicians 1.4 OB clinicians 10.3 nurses 28.4 social workers 9.9 pharmacists 12.7 phlebotomists 14.9 peer-counsellors	60.2 non-OB clinicians 6.9 OB clinicians 46.7 nurses 140.5 social workers 66.4 pharmacists 67.1 phlebotomists 99.6 peer counsellors

* Clinician calculations in table relate primarily to incremental requirements for HIV care **This category combines both ART decision-making clinicians and ART follow up care providers, which are separated in the spreadsheet model

### Scenario #2

We start year one with no one yet enrolled for HIV care. We enrol 34 000 in the first year, and grow to 68 000 individuals at the end of year two. By the end of year three, we are at 94 000 (which was the enrolment target for Mozambique by the end of 2006). See Table 3.

**Table 3:** Results of our model, Scenario 2: Starting with 34 000 enrolled for care by the end of year 1, ending with 94 000 enrolled by the end of year 3.

Clinician type*	**End year 1**	**End year 3**
Number of non-OB encounters with clinicians	7844/month	12 568/month
OB encounters with clinicians**	971/month	1226/month
FTEs required to meet this demand based on our productivity assumptions	15.7 non-OB clinicians 1.9 OB clinicians 14.0 nurses 38.7 social workers 13.6 pharmacists 17.4 phlebotomists 20.3 peer-counselors	25.1 non-OB clinicians 2.5 OB clinicians 14.3 nurses 53.0 social workers 38.3 pharmacists 28.2 phlebotomists 57.4 peer-counselors

*Clinician calculations in table relate primarily to incremental requirements for HIV care **This category combines both ART decision-making clinicians and ART follow up care providers, which are separated in the spreadsheet model

### Scenario #3

At the facility level, we model enrolling 200 individuals for care per month, starting at 0 and enrolling 4800 individuals at the end of two years, and 7200 at the end of three years (see Table 4).

**Table 4:** Results of our model, Scenario 3: Enrolling 200 patients per month, starting with zero.

Clinician type*	**End year 1**	**End year 3**
Number of non-OB encounters with clinicians	554/month	1017/month
OB encounters with clinicians**	69/month	103/month
FTEs required to meet this demand based on our productivity assumptions	1.1 non-OB clinicians 0.1 OB clinicians 1.0 nurses 2.7 social workers 1.0 pharmacists 1.2 phlebotomists 1.4 peer-counselors	2.0 non-OB clinicians 0.2 OB clinicians 1.2 nurses 4.4 social workers 2.9 pharmacists 2.3 phlebotomists 4.4 peer-counselors

*Clinician calculations in table relate primarily to incremental requirements for HIV care **This category combines both ART decision-making clinicians and ART follow up care providers, which are separated in the spreadsheet model

In the first scenario, where we have 8000 patients on ART at the end of year one and increase that number to 58 000 within two years (ending with 5753 non-OB clinician encounters per month at the end of year one, building to 30 089 by the end of the third year), we would need to increase from 11.5 non-OB clinicians to 60.2 non-OB clinicians over the period. Pharmacists would need to increase from 9.9 to 66.4 full time equivalents (FTEs).

In the second scenario where we have 34 000 patients enrolled for care at the end of year one, and increase to 94 000 by the end of year three (i.e. monthly encounters with non-OB clinicians climb from 7844 per month to 12568 per month). We would need to increase our non-OB clinician staff from 15.7 to 25.1. Notably, in this model, 10 908 patients are started on ART by the end of year one, and 36 228 by the end of year three.

In a third scenario, where we start a new clinic and enrol 200 new patients per month for two years, we would need 1.1 non-OB clinicians at the end of year one and increase to 2.0 by the end of year three. In this model, 770 patients are on ART by the end of year one, and 2738 are on ART by the end of year three.

In any scenario, the assumptions concerning ART distribution, patterns of progression of disease, proportion of patients who experience adverse drug reactions, or numbers lost to follow up will all change the FTE requirements generated. For example, an increase in the proportion of pregnant women starting ART upon enrolment (from 5% to 10%) changes scenario three from 0.2 OB clinicians to 0.4.

## Discussion

### Cautions and limitations

The numbers in our scenarios could be calculated at the facility, district or national level. When making calculations at levels above the facility, however, the user is cautioned to consider the FTE additions required to staff multiple locations. We recommend making calculations at the facility level and rounding up each facility's FTE to units that can realistically be employed (0.5 or 1.0); these individual facility numbers could then be added for district or national calculations.

Our model is intended to generate calculations for incremental workforce needs for scaling up HIV care only. Integrated care facilities will need to consider workforce needs for other health problems in addition to ART treatment. These needs will include VCT, PMTCT, tuberculosis (TB), home care, blood banks, mental health, maternity care, sexually-transmitted disease (STD) care, and inpatient care.

Our model at this point does not include administrative staff. Users should add administrative FTEs based initially on assumptions of how many fixed, baseline staff are needed for such functions as reception, medical records or data processing, human resources managers, operations managers, and the like. For example, a clinic serving 5000 patients might need 6.5 administrative staff: 1 computer support, 1 receptionist, 1 administrator, 2 janitorial, 0.4 counsellor supervisor and 1 driver. Some of these numbers would need to be increased for increases in encounter volume.

There are also training, cost and policy implications for any model that is adopted that needs further exploration.

In any exercise of this sort, the assumptions are very important in driving the conclusions. If we decrease the number of newly-enrolled patients or the number of encounters they require (demand), and/or increase the productivity of care providers either by increasing the number of hours or the number of encounters per hour (supply), then fewer staff are required to serve patient needs. Conversely, decreases in supplied staff hours or increases in encounters will drive a higher demand for health care staff. Our spreadsheet model allows for entertaining "what if" scenarios on both the demand and supply sides of the equations.

## Conclusion

We offer this modelling system as a planning tool that we hope will lead to more realistic and appropriate estimates of the workforce levels required to provide high-quality HIV care in a variety of settings. As sufficient numbers and types of health workers are brought on line at all levels – clinic, district, and national – we hope system planners will see systematic improvements in such numbers as amount of eligible patients on ART, and reductions in loss to follow up. As these numbers change, the model assumptions can be changed for the next planning cycle. Additionally, we hope users of the model will see the advantages of cross-training and task-shifting as a means to meet workforce needs.

In the case of Mozambique, as elsewhere in sub-Saharan Africa, the number of available health workers is so inadequate that the model simply illustrates the gap between what the standards of care require and the supply available to meet the need. Illustrating this gap, however, is an important step in achieving the policy goal of escalating the numbers of trained health professionals in the population [2].

## Competing interests

The author(s) declare that they have no competing interests.

## Supplementary Material

Additional file 1 **Workforce Modelling Workbook 17.xls**. Three inter-linked spreadsheets for calculating health workforce FTE requirements based on patient demand assumptions. **SUMMARY tab**. a) user-entered demand inputs and assumptions, and b) Worksheet D, which generates and displays the FTE calculations of estimated need. **ASSUMPTIONS tab**. Worksheet A maps out the schedule of encounters for each type of patient; Worksheet B counts total patient encounters generated by Worksheet A; Worksheet C generates monthly encounter counts over a three-year period from the care system's point of view. **CLINICAL ALGORITHM tab**. Describes the schedule of encounters required for each type of patient using the WHO and Mozambique treatment guidelines. Click here for file
